# A novel vertebroplasty technique using a larger-diameter needle for thoracolumbar osteoporotic vertebral compression fracture

**DOI:** 10.1097/MD.0000000000026174

**Published:** 2021-06-04

**Authors:** Eugene J. Park, Ho-Jin Lee, Min-Gu Jang, Jae-Sung Ahn, Sang Bum Kim

**Affiliations:** a Department of Orthopedic Surgery, Kyungpook National University Hospital, Kyungpook National University School of Medicine, Daegu; b Department of Orthopedic Surgery, Chungnam National University Hospital, Chungnam National University School of Medicine; c Department of Orthopedic Surgery, Konyang University Hospital, Konyang University School of Medicine, Daejeon, Republic of Korea.

**Keywords:** osteoporotic fracture, patient reported outcome measures, radiography, vertebroplasty

## Abstract

Percutaneous vertebroplasty (VP) and kyphoplasty (KP) are well-established minimally invasive surgical procedures for the treatment of osteoporotic vertebral compression fractures (OVCF). However, some drawbacks have been reported regarding these procedures, including height loss, cement leakage, and loss of the restored height after balloon deflation. We performed a novel VP technique to minimize these limitations of conventional procedures. This study aimed to compare radiological and clinical outcomes of our method using a larger-diameter needle versus conventional VP (using a smaller needle) for thoracolumbar OVCF.

From April 2016 to May 2017, 107 consecutive patients diagnosed with thoracolumbar OVCF were enrolled. Patients were divided into two groups: group 1 underwent conventional VP, i.e., using a smaller diameter needle, and group 2 underwent VP through a modified method with a larger-diameter needle. For radiological evaluation, parameters related to anterior vertebral height (AVH) and segmental angle were assessed using plain standing radiographs, and patient-reported outcomes were evaluated using the visual analog scale. Cement injection amount and leakage pattern were also analyzed. Group 2 showed a larger anterior vertebral height change than group 1 immediately postoperatively and one year postoperatively. The 1-year postoperatively-AVH maintained better in group 2 than in group 1. Group 2 showed more significant improvement of segmental angle immediately postoperatively than group 1 (3.15° in group 1 vs 9.36° in group 2). IYPo-visual analog scale significantly improved in both groups, with greater improvement in group 2 (3.69 in group 1 vs 5.63 in group 2). A substantially larger amount of cement was injected, with a lower leakage rate in group 2 than in group 1.

A novel VP technique using a larger-diameter needle showed superior radiological and clinical outcomes than conventional VP. Therefore, it can be considered a useful treatment option for OVCF.

## 1 Introduction

An osteoporotic vertebral compression fracture (OVCF) is the most common complication of osteoporosis.^[1]^ Although the majority of OVCF cases have a favorable natural history,^[2]^ some patients experience intractable pain, pulmonary dysfunction, gastrointestinal dysfunction, and sleep disorders.^[3–5]^ OVCF also causes height loss, decreased self-esteem, and depression due to kyphotic deformity,^[6]^ thus decreasing the quality of life.^[7]^ The frequency of subsequent vertebral fracture increases in proportion to the shape and severity of the deformity after the initial fracture.^[8,9]^ Therefore, surgeons should provide pain relief and attempt to prevent anterior height loss kyphosis in patients with OVCF that requires surgical intervention.^[10]^

Percutaneous vertebroplasty (VP) and kyphoplasty (KP) are well-established minimally invasive surgical procedures for the treatment of vertebral compression fractures.^[6,10,11]^ Although VP allows for rapid pain reduction in 80% to 90% of treated patients, it does not restore the vertebral height and is associated with risk for cement leakage.^[11,12]^ KP has been proposed to compensate for these shortcomings of VP and reduces pain rapidly in up to 90% of patients; moreover, it is known to restore some vertebral height with a lower risk of cement leakage.^[8,13]^ However, a significant loss of the restored height after balloon deflation, prior to cement injection, is one of the disadvantages of KP.^[11,14]^ The resulting hyperkyphotic alignment of the spine is associated with an increased risk for adjacent fractures.^[15,16]^ Furthermore, KP has some limitations, such as high cost,^[17]^ high invasiveness, and significant loss of the restored height after balloon deflation.

To overcome these disadvantages of conventional VP and KP, we performed VP through a novel technique. This technique utilizes larger-diameter Jamshidi needles with cement-filled cannulated needles for injection rather than with syringes. For this study, we hypothesized that this technique would allow a more significant amount of cement injection with minimal injury to the trabecular bone. Therefore, we aimed to compare the radiological and clinical improvements of a conventional VP with this novel VP technique.

## 2 Methods

### 2.1 Patient enrolment and management

This study was a retrospective chart review and was approved by the institutional review board of our hospital (approval no. CNUH 2019-10-004). Between April 2016 and May 2017, we enrolled patients with a diagnosis of OVCF who were admitted through the emergency department or outpatient clinic. Patients with a neurologic deficit on the lower extremities were excluded from this study. The diagnosis of recent OVCF was confirmed by magnetic resonance imaging in patients with a sudden onset of focal midline back pain. Most of the patients were diagnosed with osteoporosis (T score of −2.5 or less) on dual-energy X-ray absorptiometry and underwent related medical treatment. For those who did not fulfill the criteria of osteoporosis (T score of −2.5 or less on dual-energy X-ray absorptiometry), if they had back pain symptoms after minor trauma, such as ground-level fall or coughing, they were diagnosed with OVCF. Thoracolumbosacral orthosis was kept immediately after injury, except in a supine position. All patients who complained of persistent back pain after conservative treatment, consisting of bed rest, analgesics, external bracing, and rehabilitation, for at least 2 weeks or those who showed increasing anterior height loss on follow-up plain radiograph underwent VP. However, VP was performed immediately without 2 weeks of conservative treatment in patients who had a history of congestive heart failure, pneumonia, deep vein thrombosis, diabetes uncontrolled with an oral hypoglycemic agent, chronic kidney disease on dialysis, or aged > 80 years.

In our hospital, two attending surgeons (H.L. and J.A.) attended patients with spine conditions admitted in the emergency room alternately every other day. Both had >5 years of experience in spine surgery. One of them performed conventional VP with a smaller needle (group 1), and the other performed VP through a novel technique with a larger-diameter needle (group 2).

### 2.2 Procedure

#### 2.2.1 Operating room and patient preparation

Routinely, the operating room temperature was set at 22°C under the air-conditioning system. However, the actual operating room temperature ranged from 22.5° to 23.7°C. The humidity ranged from 12.6% to 15.8%. Patients were placed in a prone position on the operating table with either two transverse roll bars or four posts placed above and below the fractured vertebra. The bars or posts were placed to apply a distraction force to the anterior column and restore the anterior vertebral body height and lordosis. The fractured vertebrae were identified and marked under C-arm fluoroscopic guidance. Local anesthesia was performed with 2% lidocaine on the skin incision site.

### 2.3 VP with conventional diameter needle (group 1)

After a stab incision, an 11-gage (2.304 mm) Jamshidi needle (Osteo Cement Needle, JMT Co., Yangju, South Korea) was inserted through the soft tissue and pedicle to the vertebral body. After placing the Jamshidi needles bilaterally, contrast was injected to check for any extracorporeal leakage. Then, the surgeon prepared the polymethylmethacrylate (PMMA) cement (Spinofill, Injecta Co., Gunpo, South Korea) by mixing the polymer powder with monomer liquid in a bowl using a spatula. After sufficient mixing, the cement was loaded in multiple 1-ml syringes. Stepwise injection was performed by connecting the syringes to the Jamshidi needles.

### 2.4 VP with larger-diameter needle (group 2)

**Figure 1:**
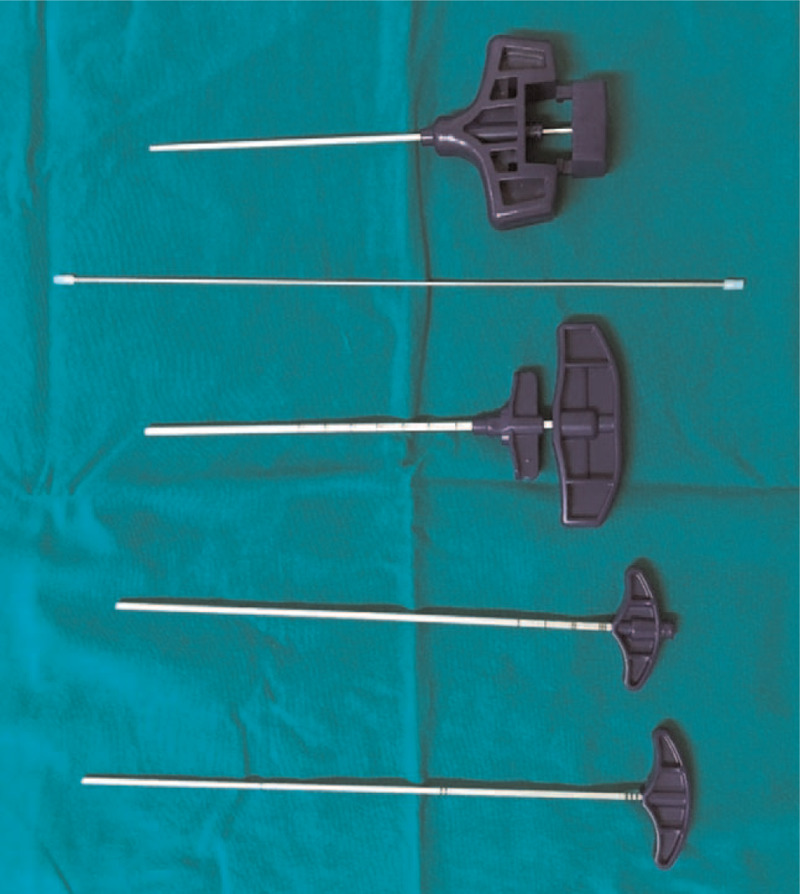
(From top to bottom) A 10-gage Jamshidi needle, guidewire, 8-gage needle connected with a stylet, 9-gage cannula, and a stylet for the 9-gage cannula.

After a stab incision, a 10-gage (2.588 mm) Jamshidi needle (Gaurdian, BM Korea Co., Gunpo, South Korea) was inserted through the soft tissue and pedicle to the vertebral body. After placing the Jamshidi needles bilaterally, the stylet was removed, and guide wires were inserted through the needles at each side. The Jamshidi needles were replaced with the 8-gage (3.263 mm) needles through the guidewire. After bilateral placement of the needles, contrast was injected to check for any extracorporeal leakage. Then, the surgeon prepared the PMMA cement (Spinofill, Injecta Co.) as mentioned above. After sufficient mixing process, the cement was loaded in multiple 9-gage (2.906 mm) cannulated needles and connected to the Jamshidi needles. The stepwise injection was performed by pushing the stylet in the cement-filled needles (Fig. 1).

### 2.5 Common technical steps in both groups

**Figure 2:**
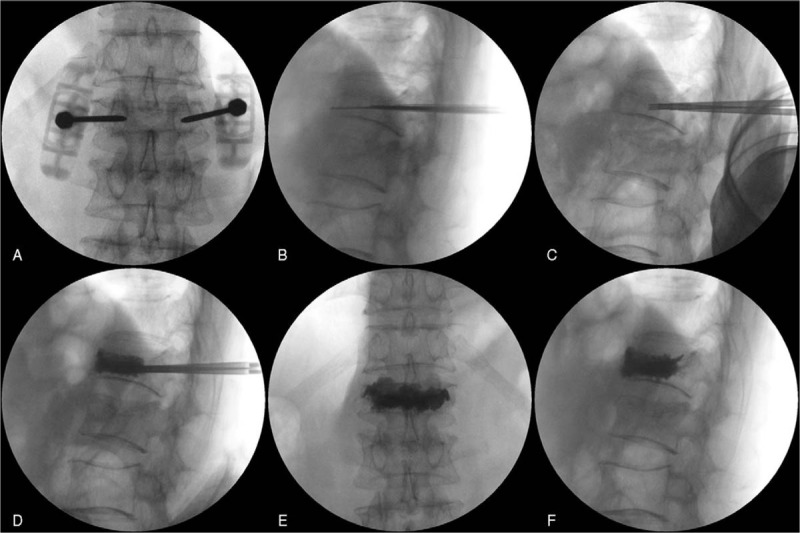
Sequential intraoperative fluoroscopic images during VP using a larger-diameter needle. (A) Bilateral placement of a 10-gage Jamshidi needle. (B) Replacing the Jamshidi needle with an 8-gage needle (dilator). (C) Insertion of the cement-loaded 9-gage cannula through the needle. (D) Cement infusion by insertion of the stylet through the 9-gage cannula. (E) Anteroposterior image after cement insertion. (F) Lateral image after cement insertion. (VP, vertebroplasty).

A fluoroscope was used throughout the procedure. The surgeon attempted to inject as much cement as possible. Injection started when the cement has a toothpaste-like consistency, yet moldable. The injection was stopped if the posterior one-third of the vertebral body started to fill on the lateral fluoroscopic image (Fig. 2).^[18]^ When the remnant cement did not smear on the gauze upon contact, Jamshidi needles were removed from the vertebral body, and the skin was sutured.

### 2.6 Clinical outcome measurements

The primary outcome measures of our study were radiological changes including the vertebral height and angle, and the rate of cement leakage. The secondary outcome parameter was Visual Analog Scale (VAS) score.

Radiological results were evaluated preoperatively, immediately postoperatively, and 1 year postoperatively using plain radiographs. The anterior vertebral height (AVH) and its changes and the segmental angle (SA) and its changes were assessed using standardized lateral plain radiographs. The AVH was measured using the anterior height of the vertebral body on the lateral plain radiographs preoperatively (Pre-AVH), immediately postoperatively (IPo-AVH), and 1 year postoperatively (1YPo-AVH). The immediate postoperative and 1-year postoperative AVH change (IPo- anterior vertebral height change [AVHC] and 1YPo-AVHC) were measured by subtracting the Pre-AVH from IPo-AVH and 1YPo-AVH. SA was measured by the angle between the upper endplate of the upper vertebra and the lower endplate of the fractured vertebra (Cobb's method) preoperatively (Pre-SA), immediately postoperatively (IPo-SA), and (1YPo-SA). A positive value indicates kyphosis, and a negative value indicates lordosis. The immediate postoperative and 1-year postoperative SA change (IPo-SAC and 1YPo- SAC, respectively) were measured by subtracting the Pre-SA from the IPo-SA and 1YPo-SA, respectively. The patient-reported outcomes were evaluated using the VAS. VAS scores were obtained preoperatively (Pre-VAS) and 1 year postoperatively (1YPo-VAS).

All statistical analyses were performed using the Statistical Package for Social Sciences (version 20.0; IBM Corp., Armonk, NY). Values are presented as mean or mean ± standard deviations, and the Kolmogorov–Smirnov test was used for the normality test. An independent *t*-test was used for the comparison of AVHC and 1YPo-SAC between the groups. A Mann–Whitney test was used for the comparison of age, IPo-SAC, VAS, cement amount, and injection time between the groups. A paired t-test was used for AVH, SA, and VAS intragroup change, except for Pre-SA and IPo-SA where Wilcoxon signed-rank test was used for comparison. Chi-square test was used to compare the sex ratio, fractured vertebral level, and cement leakage rate. Linear-by-linear association was used to compare the ratio of the number of involved levels between groups. A p-value of less than 0.05 was considered statistically significant.

## 3 Results

A total of 107 patients, with 120 affected vertebrae, were enrolled in this study, of which 64 were included in group 1 and the other 41 in group 2. In group 1, the mean age was 77.13 ± 2.3 years, 20 were male, and 44 were female, and most of the patients had one-level fracture, five had two-level fracture, and one had a three-level fracture. In group 2, the mean age was 77.26 ± 2.22 years, 7 were male, and 37 were female; besides the eight patients with two-level fracture, all patients had a one-level fracture. Group 1 consisted of 22 cases with involvement of the thoracic and 49 cases of the lumbar spine, while group 2 involved 18 cases with involvement of the thoracic and 31 cases of the lumbar spine. While other demographic data did not show difference between the two groups, the male-to-female ratio did show a significant difference (*P* = .036) (Table 1).

**Table 1:** Baseline demographic data.

	Total	Group 1	Group 2	*P*-value
No. of patients	107	64	41	
No. of vertebrae	120	71	49	
Male/female (patients)		20:44	7:34	**.036**
Age (yr) (range)		77.13 ± 2.3 (51–97)	77.26 ± 2.22 (61–93)	.928
No. of level (patients)	107			.244
- 1 level		58	33	
- 2 levels		5	8	
- 3 levels		1	0	
Level (vertebrae)	120			.513
- Thoracic		22	18	
- Lumbar		49	31	

**Figure 3:**
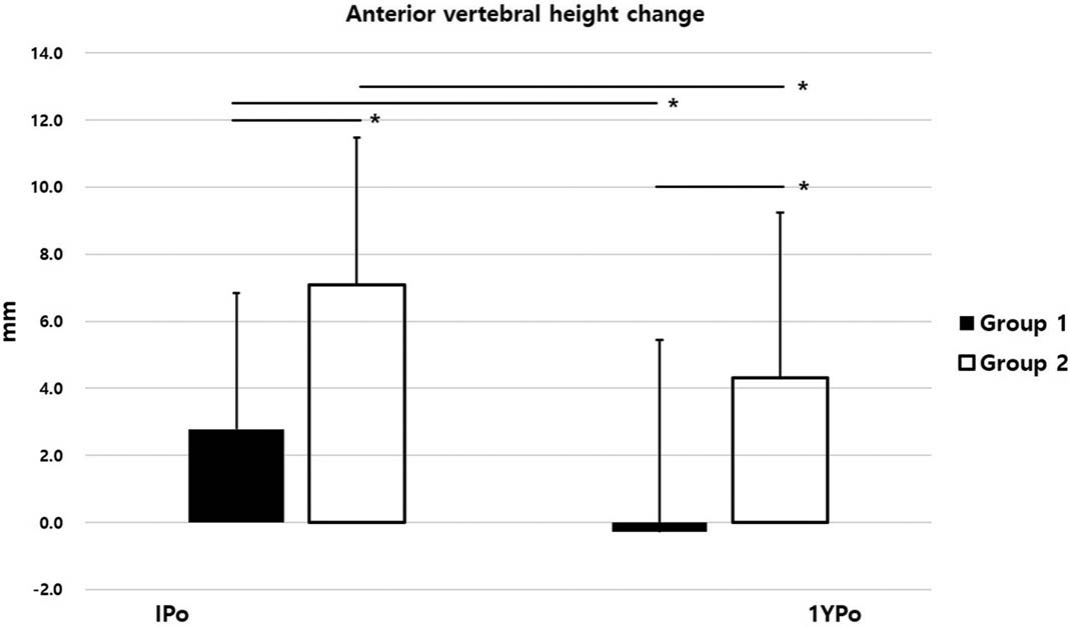
Anterior vertebral height change. (IPo, immediate postoperative; 1YPo, 1-year postoperative. ^∗^*P* < .05).

**Figure 4:**
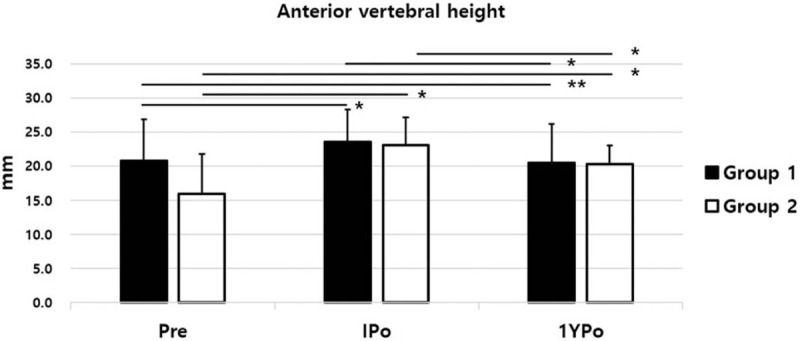
Anterior vertebral height. (Pre, preoperative; IPo, immediate postoperative; 1YPo, 1-year postoperative. ^∗^*P* < .05, ^∗∗^*P* > .05).

**Figure 5:**
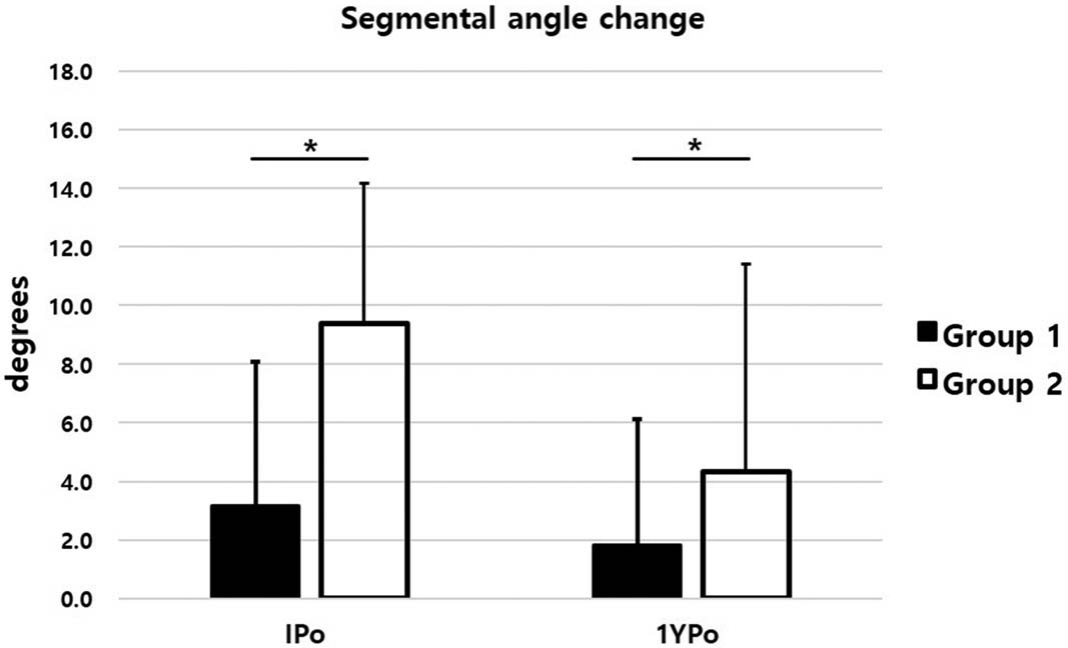
Segmental angle change. (IPo, immediate postoperative; 1YPo, 1-year postoperative. ^∗^*P* < .05).

**Figure 6:**
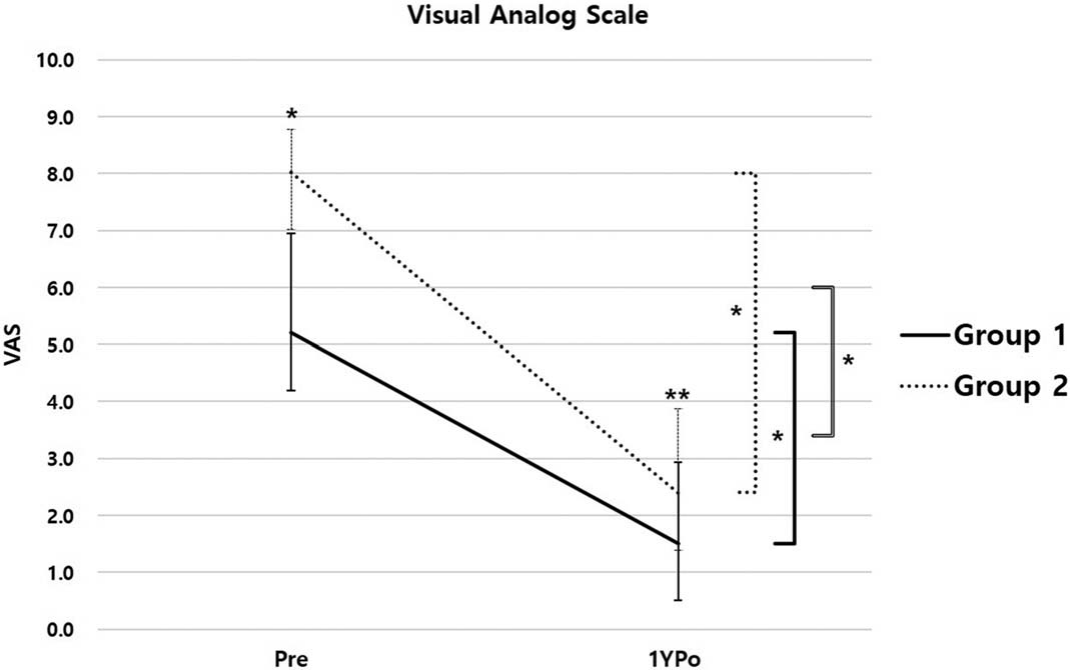
Change in VAS. Bold solid lines indicate Group 1, dotted lines indicate Group 2, and the gray line indicates the p-value of the difference between improvements in VAS scores. (VAS, visual analog scale; Pre, preoperative; 1YPo, 1-year postoperative. ^∗^*P* < .05, ^∗∗^*P* > .05).

The IPo-AVH was improved in both groups compared with Pre-AVH. The IPo-AVHC was +2.79 mm in group 1 and +7.08 mm in group 2, which showed a significant difference between the two groups (*P* = .000). The 1YPo-AVHC of group 1 was converted to a negative value of −0.28 mm, whereas the 1YPo-AVHC of group 2 remained positive at +4.32 mm (Fig. 3). The recovered AVH immediately postoperative were lost in both groups at 1 year postoperatively. However, while the 1YPo-AVH of group 1 was not different from the Pre-AVH (p = 0.588), the 1YPo-AVH of group 2 was significantly higher than the Pre-AVH (Fig. 4). In terms of SA, both groups showed significant recovery of lordosis after surgery. However, IPo-SAC were 3.15° and 9.36° in group 1 and group 2, respectively, which was significantly higher in group 2 (*P* = .000). The 1YPo-SA was significantly improved compared with Pre-SA in both groups. The 1YPo-SAC were 1.80° and 4.72° in group 1 and group 2, respectively, showing a significant difference between the two groups (Fig. 5). As regards patient-reported outcomes, the mean Pre-VAS scores were 5.20 and 8.02 in group 1 and group 2, respectively, which was significantly lower in group 1 (*P* = .000). The 1YPo-VAS significantly improved in both groups. The improvement was significantly larger in group 2 (*P* = .000) (Fig. 6).

In group 1, 5.56 ml of cement was injected 193 s after mixture, and in group 2, 6.99 ml of cement was injected 692 s after mixture. A significantly larger amount of cement was injected in group 2 at a slower pace than in group 1. A significant difference was noted in the occurrence rate of cement leakage between the two groups. group 1 had more frequent cement leakage, that is, 22 of 71 vertebrae, than group 2, that is, 6 of 49 vertebrae (p = 0.017). In group 1, 12 cases had leakage in the disk space, nine cases in the posterior aspect of the vertebral body, and one case in both the disk space and posterior aspect of the vertebral body. However, no neurologic deficit or new postoperative symptom occurred in cases with leakage posterior to the vertebral body. In group 2, leakage occurred in six of 55 cases. Four cases had leakage in the disk space and two cases in the area anterior to the vertebral body (Table 2).

**Table 2:** Comparison of cement injection details.

	Group 1	Group 2	*P*-value
Amount of injected cement (ml)	5.56 ± 1.02	6.99 ± 1.42	**.00**
Time between mixing and injection (sec)	192.6 ± 10.6	691 ± 69.9	**.00**
Extracorporeal cement leakage (patients)			**.028**
No	49	43	
Yes	22	6	
- D	12	4	
- A	0	2	
- P	9	0	
- D & P	1	0	

## 4 Discussion

Our novel VP technique has two different steps compared with the conventional technique. It uses larger-diameter Jamshidi needles and injects cement by a cement-filled cannulated needle with a stylet rather than with a 1-ml syringe. Radiological changes showed a superior outcome in terms of AVH and SA restoration and maintenance. Although the recovery of AVH and SA were lost in both groups, the group in whom this new technique was employed showed better results is terms of maintaining recovery. In addition, clinical patient-reported outcome was significantly better with the novel technique.

VP was first introduced in France for the treatment of vertebral angioma without height restoration,^[19]^ and such method has been applied for OVCF with positive results.^[12]^ The possible primary mechanism of pain relief after VP is related to the mechanical stabilization of the fractured body.^[18,20]^ However, the timing and amount of cement vary among surgeons.^[18]^ Unlike our case series, in which VP was performed 2 weeks after injury, Muijs et al. performed VP within 4–8 weeks after the fracture event and reported that it may provide not only maximum benefit but also potential risks.^[21]^ However, another study reported that severe pain caused by OVCF in an acute setting can benefit from VP, specifically within 6 weeks.^[2]^ According to a finite-element model, approximately 15% volume fraction, or 3.5 ml of cement, was needed to restore the pre-fracture stiffness, and the amount beyond such could surpass the intact state.^[18]^ Although they lacked an exact cut-off value, Ryu et al reported that the excess amount of cement could increase the incidence of cement leakage.^[22]^ Our result showed that a large amount of cement injected was associated with a lower occurrence rate of cement leakage, which is assumed to be due to lower injection pressure when using a larger needle than when using a conventional (smaller) needle.

Vertebral body collapse after a vertebral compression fracture eventually causes hyperkyphotic deformity, which can be more pronounced in proportion to the number and severity of vertebral compression fractures, especially wedge type. In addition, the excessive kyphotic deformity may cause decreased pulmonary function and increased risk of subsequent fracture risks.^[15,16]^ Since VP was initially performed without consideration of the anterior height restoration of the vertebral body,^[19]^ KP including the ballooning process was introduced. Although Dong et al. reported that KP leads to better kyphotic angle correction than VP, the clinical outcome was significantly improved in both techniques.^[12]^ On the contrary, a cadaveric study concluded that both VP and KP reduces the spine segment significantly.^[8]^ However, KP has some disadvantages, such as high cost instruments, raising the operation fee.^[23,24]^ In addition, KP is an invasive procedure since it requires a balloon inflation process and general anesthesia in some cases.^[11,25]^ The ballooning process creates a void for the injected cement to avoid leakage. However, it may induce a re-collapse at the index level, which is correlated with poor clinical outcome. Such complications seldom occurred with VP.^[24]^ Moreover, stentoplasty has been used to solve such problems by keeping the stent inside the cavity after deflation of the balloon, preventing re-collapse.^[11]^ Although stentoplasty showed less reduction than KP, since it includes the ballooning process and keeps the stent inside the vertebral body, increased invasiveness is still a problem. In a systematic review, compared with VP, stentoplasty has some advantages, including restoration of kyphotic change and bone cement volume. Still, barely limited evidence supports the improved height restoration or clinical outcome.^[25]^ Flors et al. reported vesselplasty as an alternative technique, which used a porous structured polyethylene terephthalate balloon bone cement that permeated through its wall. However, only a minimum increment of vertebral height was noted postoperatively.^[20]^ Our technique lacks the balloon inflation process and general anesthesia is not required. The cost of the procedure was also lower than that of KP since it does not use any disposable instruments for ballooning, which mainly accounts for the cost of KP.^[17]^ According to Ahn et al, VP using a larger-diameter filler can inject more PMMA cement and superior to conventional VP for vertebral height restoration or kyphotic angle correction.^[26]^

Possible advantages of our procedure compared with KP and conventional VP are as follows. The normal bone trabeculae of the vertebral body could be preserved since the PMMA cement was injected without making a void by additional balloon dilatation compared with KP, thus minimizing collapse during follow-up. Since our procedure did not include any ballooning procedure, intractable intraoperative pain could be avoided in some patients who undergo KP. Compared with a conventional 11-gage Jamshidi needle, if the surgeon applies the same injecting force, a 9-gage needle increases the surface area by approximately 20% and subsequently decreases the pressure, according to the following equation:

p=F/A

(*p*: pressure, *F*: force, *A*: surface area)

The decreased injection pressure may avoid disruption of the microstructure of the cancellous bones and cement leakage. The present technique also enables injection of higher amount of cement with increased thickness due to the larger diameter of the needle, which may have led to increased height recovery and less cement leakage. Our results suggest that using a needle with larger-diameter as possible may be beneficial. Lee et al. reported the importance of anterior height restoration immediately after surgery, especially in severely collapsed vertebrae, although the restoration can be lost during follow-up.^[10]^

This study has some limitations. First, results are limited given the retrospective nature of this study. Second, although we have synchronized the operating room temperature setting, the actual values were not identical. The temperature difference might have influenced the polymerization time, viscosity, and timing of injection, and this varied greatly in group 2. Third, the male-to-female ratio and preoperative VAS were significantly different between the two groups probably due to small sample size. Forth, follow-up period of 1 year was relatively short to enable generalization of the long-term results of this technique. Future prospective randomized controlled trials with longer follow-up are necessary. Finally, since the procedure for each group was performed by different surgeon, selection bias cannot be excluded. However, the inclusion criteria for vertebroplasty were identical in both groups to minimize the bias.

This novel VP technique using a larger-diameter needle for OVCF may improve the radiological outcome, including AVH and SA, and patient-reported outcome. This technique yielded superior radiological and clinical results to the conventional method with less cement leakage rate. It can be considered an effective treatment option for patients with thoracolumbar OVCF.

## Acknowledgments

The authors would like to thank Enago for the English language review.

## Author contributions

**Conceptualization:** Ho-Jin Lee, Sang Bum Kim.

**Data curation:** Eugene J. Park.

**Investigation:** Eugene J. Park, Ho-Jin Lee, Min-Gu Jang.

**Methodology:** Ho-Jin Lee, Jae-Sung Ahn.

**Project administration:** Eugene J. Park, Min-Gu Jang.

**Software:** Eugene J. Park.

**Supervision:** Ho-Jin Lee, Jae-Sung Ahn.

**Validation:** Jae-Sung Ahn.

**Visualization:** Eugene J. Park, Sang Bum Kim.

**Writing – original draft:** Eugene J. Park.

**Writing – review & editing:** Eugene J. Park, Ho-Jin Lee, Min-Gu Jang.
